# Transmission Electron Microscopic Morphological Study and Flow Cytometric Viability Assessment of *Acinetobacter baumannii* Susceptible to *Musca domestica* cecropin

**DOI:** 10.1155/2014/657536

**Published:** 2014-05-05

**Authors:** Shuiqing Gui, Rongjiang Li, Yongwen Feng, Sanming Wang

**Affiliations:** ^1^Intensive Care Unit, Shenzhen Second People's Hospital, Shenzhen, Guangdong 518031, China; ^2^Department of General Surgery, Xixiang People's Hospital, Shenzhen, Guangdong 518102, China; ^3^Department of Vascular Surgery, Guangdong General Hospital, Guangdong Academy of Medical Sciences, Guangzhou, Guangdong 510080, China

## Abstract

Multidrug-resistant (MDR) *Acinetobacter baumannii* infections are difficult to treat owing to the extremely limited armamentarium. Expectations about antimicrobial peptides' use as new powerful antibacterial agents have been raised on the basis of their unique mechanism of action. *Musca domestica* cecropin (Mdc), a novel antimicrobial peptide from the larvae of Housefly (*Musca domestica*), has potently active against Gram-positive and Gram-negative bacteria standard strain. Here we evaluated the antibacterial activity of Mdc against clinical isolates of MDR-*A. baumannii* and elucidate the related antibacterial mechanisms. The minimal inhibitory concentration (MIC) of Mdc was 4 **μ**g/mL. Bactericidal kinetics of Mdc revealed rapid killing of *A. baumannii* (30 min). Flow cytometry using viability stain demonstrated that Mdc causes *A. baumannii* membrane permeabilization in a concentration- and time-dependent process, which correlates with the bactericidal action. Moreover, transmission electron microscopic (TEM) examination showed that Mdc is capable of disrupting the membrane of bacterial cells, resulting in efflux of essential cytoplasmic components. Overall, Mdc could be a promising antibacterial agent for MDR-*A. baumannii* infections.

## 1. Introduction

*Acinetobacter baumannii* (*A. baumannii*) has been recognized as one of the most problematic pathogen threat to public health in recent years [1, 2]. Particularly, many strains of* A. baumannii* have become resistant to common antibiotics, including aminoglycosides, fluoroquinolones, tetracyclines, carbapenems, and other extended-spectrum *β*-lactams, through a variety of mechanisms [3–6]. Apart from its intrinsic resistance mainly due to constitutive expression of certain efflux pumps as well as the low permeability of the outer membrane to certain antibiotics,* A. baumannii* is able to easily acquire and incorporate genetic elements such as transposons, integrons, and plasmids [3, 7–9]. Nosocomial infections, particularly in intensive care units, due to multidrug-resistant (MDR) isolates of* A. baumannii* are associated with increased morbidity and mortality. Therefore, an increased effort to search for new antimicrobial agents with antibacterial mechanisms that differ from common antibiotics is required.

Antimicrobial peptides (AMPs) have been isolated from a wide range of insects, bacteria, vertebrates, and plants [10]. They play one of the most important roles against pathogenic microorganisms in host defense system [11, 12]. In contrast with most antibiotics, AMPs generally exert their antimicrobial effect through physical interactions with cell membrane of target organisms [13]. This unique mechanism of action may reduce likelihood of emergence of resistance, thus raising the expectations about antimicrobial peptides' use as new powerful antimicrobial agents. A significant number of AMPs have been investigated against multidrug- resistant isolates both in vitro and in vivo [14, 15]. Many of these belong to the family of cecropin peptide [16]. Previous studies in cecropin and its related peptides demonstrate that formation of membrane-spanning pores that disrupt the cell membrane of the bacteria seems to be the most likely mechanism [17, 18]. However, the details surrounding the mechanism of bactericidal still need to be elucidated.

*Musca domestica* cecropin (Mdc) has been identified and characterized from the larvae of Housefly (*Musca domestica*), which has been used clinically to cure osteomyelitis, decubital necrosis, lip boil, and ecthyma and others in China [19]. The peptide exerts a potent antibacterial effect against Gram-negative and Gram-positive bacteria standard strain [20]. A limitation for the transition of cecropin to therapeutic purpose is the hemolytic or cytotoxicity properties [21]. Of important note, Mdc did not show a perceptible cytotoxic effect on human red blood cells [22]. So, it is a subject for further studies whether Mdc will be a novel and effective clinical alternative against the increasing threat of a widespread dissemination of multidrug-resistant* A. baumannii*. The aim of the present study was to evaluate the antibacterial activity of Mdc against clinical isolates of multidrug-resistant nosocomial isolates of* A. baumannii*. We also attempted to acquire the details surrounding the antibacterial mechanism by flow cytometry and transmission electron microscopy.

## 2. Materials and Methods

### 2.1. Reagents

*Musca domestica* cecropin (GWLKKIGKKIERVGQHTRDATIQTIGVAQQAANVAATLKG-NH_2_) was prepared by conventional Fmoc solid-phase synthetic method with a 431 peptide synthesizer (Applied Biosystems Inc., Foster City, CA). The synthesized peptide was purified to near homogeneity (>95%) by preparative reversed phase-high performance liquid chromatography (RP-HPLC) (Waters Delta-Pak C18, 15 *μ*m, 300 Å, 25 × 100 mm) and was further characterized by analytical RP-HPLC (Waters Symmetry C18, 3.5 *μ*m, 100 Å, 4.6 × 150 mm) and mass determination of the eluate with an API electrospray ionization mass spectrometer (Perkin Elmer SCIEX). All other chemicals used were of analytical grade.

### 2.2. Microorganisms and Medium

Multidrug-resistant clinical isolate of* A. baumannii* GIM1.650 was obtained from the Center of Medical Laboratory of the First Affiliated Hospital of Guangdong Pharmaceutical University, Guangzhou, China. This strain is resistant to most tested antibiotics including ampicillin (>16 *μ*g/mL), cefazolin (>16 *μ*g/mL), ceftriaxone (>32 *μ*g/mL), ceftazidime (>16 *μ*g/mL), piperacillin (>64 *μ*g/mL), cefepime (>16 *μ*g/mL), aztreonam (>16 *μ*g/mL), ampicillin/sulbactam (>16 *μ*g/mL), amoxicillin/clavulanic acid (>16 *μ*g/mL), piperacillin/tazobactam (>64 *μ*g/mL), gentamicin (>8 *μ*g/mL), ciprofloxacin (>2 *μ*g/mL), amikacin (>32 *μ*g/mL), levofloxacin (>32 *μ*g/mL), imipenem (>8 *μ*g/mL), meropenem (>8 *μ*g/mL), Tetracycline (>8 *μ*g/mL), bactrim (>2 *μ*g/mL), and levofloxacin (>8 *μ*g/mL) with the exception of polymyxin E (⩽0.5 *μ*g/mL). The control strains* A. baumannii* ATCC 19606 were obtained from American Type Culture Collection (ATCC). The compositions of medium in this work are as follows: Luria-Bertani (LB) medium (*w/v*): 1% NaCl, 1% tryptone, 0.5% yeast extract and Mueller-Hutton (MH) medium (*w/v*): 1.75% casamino acid, 0.5% beef extract, and 0.15% starch. LB medium was used for preincubation of the test bacteria. MH medium was employed for antibacterial assays.

### 2.3. Antimicrobial Activity Assay

For determination of minimal inhibitory concentration (MIC), the broth microdilution method for Mdc was carried out according to the procedures outlined by the Clinical and Laboratory Standards Institute. Briefly, test bacteria strains were inoculated to 5 mL of MH broth (MHB), then grown overnight to OD_600 nm_ of 0.8 with shaking (200 rpm), and diluted to 2 × 10^6^ colony forming units (CFUs) per mL with MHB medium. A 100 *μ*L cell suspension was dispensed in each well of sterile 96-well polypropylene microtiter plate (Sigma-Aldrich), and 100 *μ*L of MHB without bacterial suspension was served as negative controls. A 100 *μ*L of each 2-fold serial dilutions in phosphate-buffered saline (PBS) solution was prepared from a stock solution of the peptide (128 *μ*g/mL), and PBS was used as positive controls. The plate was incubated for 18–24 h at 37°C, and the MIC was defined as the lowest concentration of antimicrobial agent that completely inhibits growth of the organism in the microdilution wells measured by turbidimetry at 600 nm. The results were present as mean values of three independent experiments.* A. baumannii *ATCC 19606, a reference strain that is susceptible to colistin sulfate, was used as a control. Minimal bactericidal concentration (MBC) was recorded as the lowest concentration of peptide that killed 99.9% of the test inoculum by viable counting assay. To identify the growth inhibitory activity kinetic, OD_600 nm_ of 96-well plate prepared as above was monitored over time until 16 h stationary incubation at 37°C.

### 2.4. Kinetics of Bacterial Killing

Kinetics of bacterial killing was determined according to the method described in Lu et al. [22]. Log-phase* A. baumannii* strains GIM1.650 (10^6^ CFU/mL) were incubated with culture medium containing zero or MIC, 1/4 MIC Mdc for 120 min. Aliquots of the mix were removed at fixed intervals, serially diluted 10-fold in PBS, plated on LB agar, and incubated 16–24 h at 37°C. CFU was counted to determine cell viability. PBS solution was used as the control. The experiments were carried out in duplicate.

### 2.5. Membrane Permeabilization by Flow Cytometry Analysis

Membrane permeabilization of Mdc on bacterial was investigated by flow cytometry using the DNA intercalating dye propidium iodide (PI).* A. baumannii* strains GIM1.650 strains were prepared as in Section 2.3. A suspension of approximately 2 × 10^6^ cells per mL at log phase was treated with Mdc (0–2 × MIC) Mdc and incubated at 37°C for 0–120 min. The bacterial cells were harvested by centrifugation and stained with PI (Invitrogen Ltd, Paisley, UK) (final concentration 5 mg/mL) at room temperature in the dark for 30 min. Flow cytometry was performed using a FACScan (BD Biosciences, NJ, USA). Bacteria were initially gated using forward scatter (FS) and then analyzed for red fluorescence. All experiments were conducted in triplicate and for each sample 10 000 stained bacteria were recorded. Heat-killed cells (at 70°C for 30 min) were used as a positive control of PI and bacteria without Mdc were used as a viability control.

### 2.6. Transmission Electron Microscopy

Transmission electron microscopy (TEM) was used to evaluate the morphological changes in* A. baumannii* cells after treatment with Mdc.* A. baumannii* cells in log phase being collected and incubated for 60 min at 37°C in the presence and absence of the Mdc at a concentration of 8 *μ*g/mL. Upon incubation, the pellet obtained after centrifugation was fixed in 0.1 M phosphate buffer (containing 3% of glutaraldehyde and 1.5% paraformaldehyde) for 2.5 h at 4°C. To postfix the cells, 1% osmium tetroxide and 1.5% potassium ferrocyanide were added and the samples were left at 4°C for 1.5 h. The samples were dehydrated in an ethanol series and embedded in epoxy resin. Thin slices of the pellet were made and stained with 2% uranyl acetate for 1 h. The sections were examined in a JEM1400 (Jeol, Tokyo, Japan) transmission electron microscopy and microphotographs were taken with a digital camera Bioscan 792 (Gatan Inc., Pleasanton, USA).

## 3. Results

### 3.1. Antimicrobial Activity

To test for antimicrobial activity of Mdc, MIC and MBC were determined by broth microdilution method. The results of MIC (4 *μ*g/mL) and MBC (8 *μ*g/mL) did not show significant differences among the control strain ATCC 19606 and the multidrug-resistant clinical isolate of* A. baumannii *(Table 1).

### 3.2. Kinetics of Bacterial Killing

**Figure 1:**
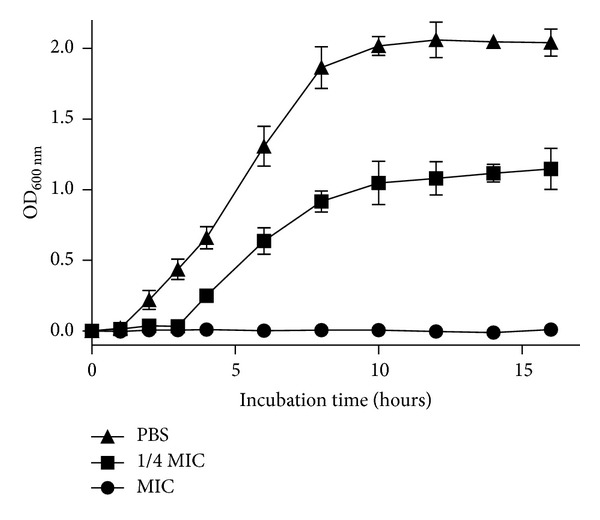
Growth inhibitory activity of Mdc against* A. baumannii *GIM1.650. The experiment was carried out in triplicate, and plots represent the average values. Error bars refer to the standard deviations.

**Figure 2:**
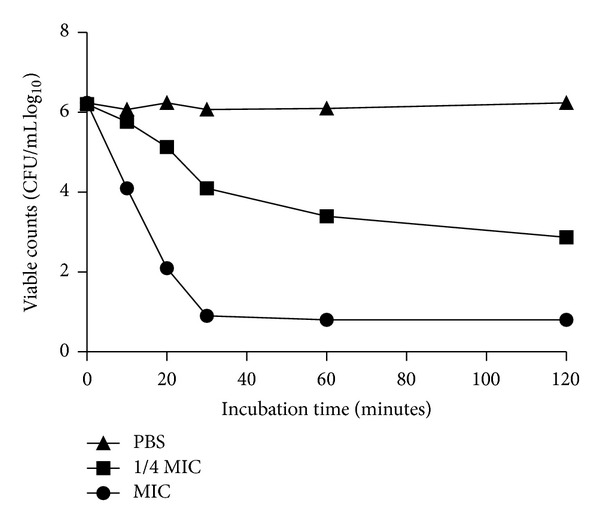
Bactericidal kinetics of Mdc against* A. baumannii* GIM1.650 after incubation with different concentration of Mdc. A 5-log reduction occurs after 30 min at the MIC (4 *μ*g/mL). The experiment was carried out in triplicate, and average values are reported (SD ⩽10%).

The growth inhibitory activity kinetic was investigated and the kinetic plot is shown in Figure 1. The MIC (4 *μ*g/mL) completely inhibited* A. baumannii *GIM1.650 growth. Below the MIC (1 *μ*g/mL), the growth lag was greater than the zero peptide control. To further explore its antimicrobial potency of Mdc, kinetics of bacterial killing were conducted to determine the rate of lysis. The results showed that Mdc reduced the* A. baumannii *GIM1.650 CFU at concentrations of MIC (4 *μ*g/mL), 1/4 MIC (1 *μ*g/mL) after 10 min incubation compared with negative controls. At concentrations of MIC (4 *μ*g/mL), killing by Mdc was complete after a 30 min exposure period (Figure 2).

### 3.3. Membrane Permeabilization by Flow Cytometry Analysis

**Figure 3:**
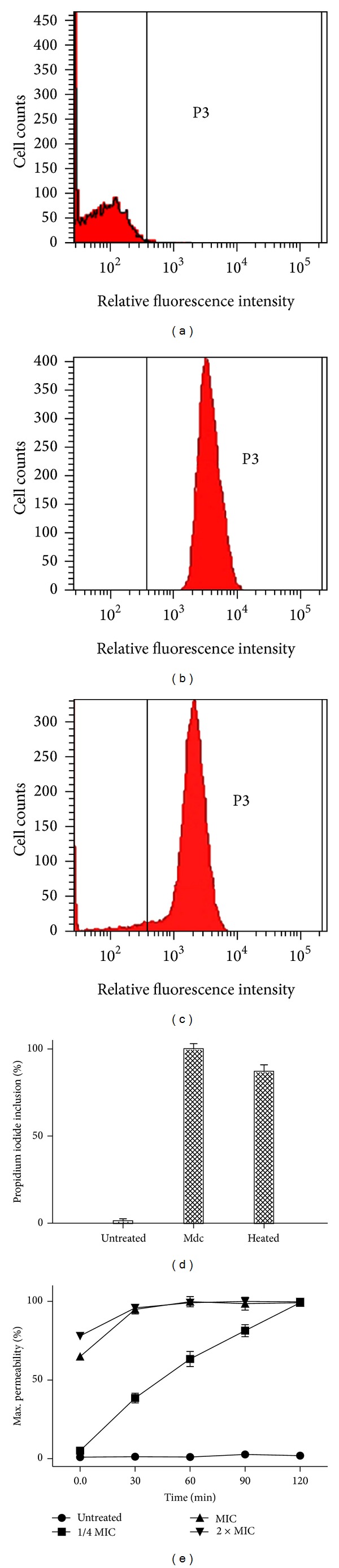
Effect of Mdc on the membrane permeability of* A. baumannii* GIM1.650 analyzed by the flow cytometer. The relative fluorescence intensities within the P3 regions were taken as PI-positive cells. (a) Untreated cells control, (b) cells treated with 4 *μ*g/mL Mdc at 37°C for 1 hour, (c) dead control (cells heated at 70°C), (d) percentage of cells stained with propidium iodide (PI), and (e) kinetics of Mdc membrane permeability against* A. baumannii.*

To determine whether Mdc has an effect on the integrity of bacterial membrane, we tested its effect on bacterial cells by PI-based flow cytometry analysis. Flow cytometric analysis of* A. baumannii* viability control showed that the bacteria were 98.89% viable (Figure 3(a)). The positive control showed that a total of 86.87% of heat treated cells were permeable to PI (Figure 3(c)). It was interestingly found that following incubation with Mdc at MIC result in 99.78% of* A. baumannii* cells were PI-positive cells (Figure 3(b)). Kinetics of Mdc membrane permeability against* A. baumannii *was shown to be a time- and concentration-dependent process (Figure 3(e)).

### 3.4. Transmission Electron Microscopy (TEM)

**Figure 4:**
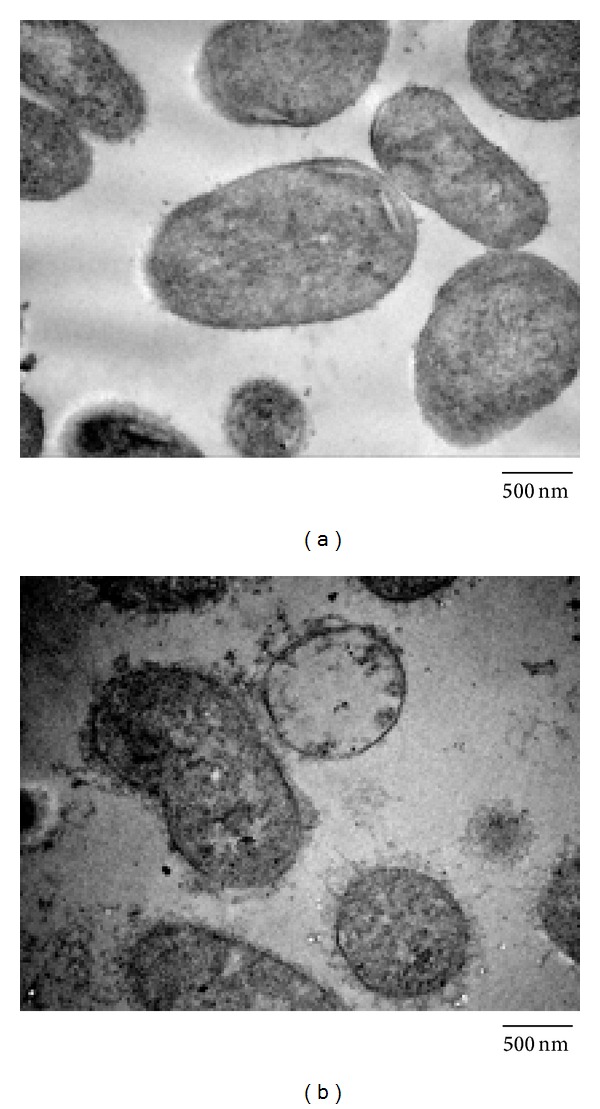
Ultrastructural damages in* A. baumannii* GIM1.650 treated with Mdc. Bacteria were untreated (a) or treated with Mdc (b) at a concentration of 8 *μ*g/mL. Cells were processed to be analyzed under TEM.

To investigate the morphological changes of* A. baumannii* cells after being treated with Mdc, transmission electron microscopy was used. The intact (control) cells of* A. baumannii* were shown in Figure 4(a), in which the inner and outer membranes of the* A. baumannii* envelope were smooth. The cytoplasmic content of bacterial cell was evenly distributed and the cell aggregation was not detected. Compared with control cells, significant morphological changes were observed in Mdc treated* A. baumannii* cells (Figure 4(b)). A great number of cells appeared with their cell membrane damaging and the cytoplasm content leaked to the extracellular medium. The outer membrane of the bacteria cell is distended and displaced in relation to the plasma membrane. Furthermore, coagulated material in cytoplasm was observed.

## 4. Discussion

*A. baumannii* acts as an opportunistic pathogen, causing severe nosocomial infections with high mortality rates [2]. The outstanding capacity of* A. baumannii* to develop resistance against the common antibacterial agents [23] makes this pathogen as important target for bactericidal activity evaluation of Mdc. In the present study, the same MIC and MBC value of Mdc were found in control strain ATCC 19606 and multidrug-resistant clinical isolate GIM1.650. The results indicate that Mdc has potent antibacterial activity against* A. baumannii*, and its mechanism of antimicrobial action is different from the common antibacterial agents. The lag time and an obvious slower growth rate shown in Figure 1 indicating cell growth have been inhibited by Mdc initial at 1 *μ*g/mL, but the interaction was not sufficient to kill the cells. Killing kinetic analysis was conducted to further explore the antimicrobial action of Mdc at the MIC, 1/4 MIC concentration. The results revealed that Mdc can mediate rapid killing of MDR-*A. baumannii* in 30 min and resulted in log orders of cell complete lysis at a concentration as low as 4 *μ*g/mL (Figure 2). These observations were in agreement with previous results obtained in other multidrug-resistant strains of bacteria with antimicrobial peptides [17].

A number of techniques are available for studying the mechanisms of antimicrobial action on bacteria [13, 24, 25]. In the present work, we set up a study using different tools (flow cytometry and transmission electron microscopy) to address the same hypothesis. At first, to investigate the membrane permeability of MDR-*A. baumannii*, PI-based flow cytometry assay was performed to determine bacterial viability. PI is a specific nucleic acid-binding dye that is fully excluded from intact bacterial cells. When the membrane of the bacteria is compromised, the dye penetrates the cell and binds readily to nucleic acids, thus selectively labeling dead cells. The results of flow cytometry assay (Figures 3(b) and 3(d)) indicate that the bacterial membrane is permeabilized by Mdc. To explore a more definitive correlation of membrane permeability and bactericidal action, the kinetics of membrane permeability was assayed (Figure 3(e)). The results showed that membrane permeability of* A. baumannii *by Mdc is a time- and concentration-dependent manner. At the MIC of 4 *μ*g/mL, the maximum permeability is achieved at 30 min. Above the MIC, the extent of permeability did not change. Below the MIC (1 *μ*g/mL), it takes 2 h to approach the maximum permeability. The rate of permeability for 4 *μ*g/mL and 8 *μ*g/mL is similar, whereas 1 *μ*g/mL is slightly faster. Bactericidal kinetics showed complete killing of* A. baumannii* in 30 min by Mdc at MIC (Figure 1), which directly correlates with the permeabilization. This suggests that, at and above the MIC, the immediate permeabilization caused damage too significant for the cell to recover and resume growth. Blow the MIC (1 *μ*g/mL), Mdc appears capable of causing disruptions to the membrane, resulting in damaged cells that subsequently recover.

Second, in order to further investigate the effect of Mdc on cell membrane, we observed the Mdc treated cells using TEM. The results showed that Mdc is capable of disrupting the membrane of sensitive cells, resulting in efflux of essential cytoplasmic components. Moreover, the outer membrane is displaced and distended with the inner membrane, possibly due to the disruption of contact sites between outer membrane and peptidoglycan or the increasing number of Mdc-induced pores that cause the increasing hydrostatic pressure in the periplasmic space.

In conclusion, the results of the present study demonstrated that Mdc has potent antibacterial activity against multidrug-resistant nosocomial isolates of* A. baumannii*. Bactericidal kinetics, flow cytometry, and transmission electron microscopic assays indicated that Mdc could alter the permeability properties to mediate the disruption of* A. baumannii* membrane, which then leads to the death of bacterial cells. Overall, Mdc could be a novel and promising clinical alternative against the increasing threat of a widespread dissemination of multidrug-resistant* A. baumannii.*

## Figures and Tables

**Table 1:** Minimal inhibition concentration (MIC) and minimum bactericidal concentration (MBC) of Mdc.

	MIC (*μ*g/mL)	MBC (*μ*g/mL)
ATCC 19606	4	8
GIM 1.650	4	8
